# Measurement of the α1-proteinase inhibitor (α1-antitrypsin) of common marmoset and intestinal protein loss in wasting syndrome

**DOI:** 10.1042/BSR20190562

**Published:** 2019-07-08

**Authors:** Kimie Niimi, Hiromasa Morishita, Masaya Usui, Reiko Ito, Shino Kurata, Nobuko Mataga, Eiki Takahashi

**Affiliations:** 1 Support Unit for Animal Resources Development, Research Resources Division, RIKEN Center for Brain Science, 2-1 Hirosawa, Wako-shi, Saitama 351-0198, Japan; 2 Support Unit for Bio-Material Analysis, Research Resources Division, RIKEN Center for Brain Science, 2-1 Hirosawa, Wako-shi, Saitama 351-0198, Japan

**Keywords:** alpha1-proteinase inhibitor (α1-PI), common marmoset, intestinal protein loss, wasting marmoset syndrome

## Abstract

Although wasting marmoset syndrome (WMS) is one of the biggest problems facing captive marmoset colonies, the mechanisms underlying its pathogenesis remain unclear. In our clinical experience, it is difficult to cure WMS-affected marmosets with severe hypoalbuminemia. Thus, the mechanisms underlying hypoalbuminemia in WMS must be understood. In the present study, we investigated whether intestinal protein loss, a known reason for hypoalbuminemia, occurs in this disease. Fecal α1-proteinase inhibitor (α1-PI, also known as α1-antitrypsin) has been used to diagnose intestinal protein loss in other species. To develop an assay system for this protein, marmoset α1-PI was purified from plasma and antibodies against it were developed using the purified protein. Using the antibodies, a sandwich enzyme-linked immunosorbent assay (ELISA) to measure marmoset α1-PI was developed, and its detection sensitivity for fecal samples was ∼20-fold higher than that of a commercial kit for human α1-PI. From this ELISA, the reference intervals for serum and feces of healthy marmosets were 0.87–1.85 mg/ml and 0.53–395.58 μg/g, respectively. The average concentrations of α1-PI in serum and feces of seven WMS-affected marmosets were 1.17 mg/ml and 1357.58 μg/g, respectively. Although there were no significant differences in the serum concentrations between healthy and WMS-affected marmosets, the fecal concentrations were significantly higher in WMS-affected marmosets than in healthy individuals, suggesting that intestinal protein loss occurs in WMS. Intestinal protein loss of WMS-affected marmosets was significantly attenuated with treatment, suggesting that it is one of the mechanisms involved in the hypoalbuminemia observed in WMS.

## Introduction

The common marmoset (*Callithrix jacchus*) is a new world monkey native to northern and eastern Brazil. Compared with old world monkeys, they have several advantages including small body size, ease of handling, ease of breeding in captivity, and absence of severe zoonotic issues [1]. For these reasons, common marmosets have been used as experimental animals in many fields such as reproductive biology, drug development, infectious disease, and studies of the brain [1–7]. In addition, production of genetically modified marmosets has become possible in recent years [8,9].

Wasting marmoset syndrome (WMS) is a unique disease in this species and is one of the most serious problems in the management of common marmosets. The main symptoms are weight loss, decreased muscle mass, anemia, hypoalbuminemia, and chronic diarrhea [10,11]. Some studies have reported that 60.5% of captive marmosets [11] suffer from WMS, and 31–44% of deaths [12] involve this illness. Although WMS is one of the biggest problems in maintaining captive marmoset colonies, the mechanisms of pathogenesis remain unclear. There are a few effective treatments for WMS [13,14]; however, in our clinical experience, using tranexamic acid for marmosets with serum levels of albumin (Alb) < 2.0 g/dl has failed to cure WMS. Thus, the mechanisms underlying hypoalbuminemia observed in WMS must be understood to advance the treatment for this disease.

The causes of hypoalbuminemia include infection, trauma, cancer, nephrotic syndrome, and protein-losing enteropathy [15]. Although infection of *Trichospirura leptostoma* has been reported to cause WMS [16], infectious diseases have been excluded as causes of hypoalbuminemia observed in WMS because not all WMS-affected marmosets have infections. Trauma and cancer are also not relevant to WMS and no symptoms of nephrotic syndrome have been observed in WMS. Thus, in the present study, we investigated whether intestinal protein loss occurred in WMS.

To detect intestinal protein loss, measurements of fecal α1-proteinase inhibitor (α1-PI) have been conducted in humans [17,18], dogs [19,20], and cats [21]. α1-PI is a serum glycoprotein synthesized by the liver [22] and released into the systemic circulation, and is involved in the neutralization of proteolytic enzymes to protect various tissues from damage [23]. Under physiological conditions, it is rarely found in the lumen of the gastrointestinal tract. Because α1-PI has a similar molecular weight as Alb, it is lost to the gastrointestinal tract at a rate comparable with that of Alb [24]. However, unlike Alb, α1-PI is resistant to bacterial degradation and the effects of digestive enzymes in the lumen of the gut, enabling it to be detected in fecal samples by immunoassay [20]. Purification and characterization of marmoset α1-PI were reported by Parambeth et al. [24]. However, measurements of marmoset α1-PI have never been reported.

In the present study, we developed an immunoassay to measure α1-PI levels in serum and fecal samples and compared the concentrations between samples from healthy marmosets and WMS-affected individuals.

## Materials and methods

### Animals

The present study was approved and overseen by the Animal Experiments Committee of RIKEN (Saitama, Japan), and was conducted in accordance with the Institutional Guidelines for Experiments using Animals. Common marmosets were reared at the RIKEN Center for Brain Science (Saitama, Japan), and maintained on a 12-h light–dark cycle at 27°C and 50% humidity. All marmosets in the present study were 2–8 years old. Marmosets were allowed *ad libitum* access to water and food pellets (CMS-1 M; Clea Japan Inc., Tokyo, Japan) with added vitamins C and D, calcium, and acidophilus. Hot water and comb honey were also added to soften the pellets and improve the animals’ preference for the food. Animals were given pieces of castella (Yamazaki Baking Co., Ltd., Tokyo, Japan) or banana pudding (Kewpie Co., Tokyo, Japan) as treats.

### Affinity chromatography by α1-antitrypsin select resin

Marmoset pooled plasma was diluted with a binding buffer (20 mM Tris/HCl with 50 mM NaCl, pH 7.4) at a ratio of 1:9. The diluted plasma was filtered through a 0.45-μm filter (GL Science, Japan) and added to α1-antitrypsin select resin (GE Healthcare Life Science, Tokyo, Japan), which was equilibrated with the binding buffer. For the batch purification step, the plasma with the resin was shaken at 4°C for 10 min and the resin-captured α1-PI was packed in a Glass Econo-Column (φ10 mm × 100 mm; Bio-Rad) coupled with the ÄKTA 10s system. The column was washed with the binding buffer and α1-PI contained fractions were eluted and collected in 1 ml fractions with an elution buffer (20 mM Tris/HCl with 2 M MgCl_2_, pH 7.4) at a flow rate of 1 ml/min.

### Gel filtration chromatography

The α1-PI containing fractions from the affinity chromatography were directly loaded on to a HiLoad 16/60 Superdex 200 pg column (GE Healthcare Life Science), which was equilibrated with 20 mM Tris/HCl buffer containing 50 mM NaCl, pH 7.4 at a flow rate 0.5 ml/min. A 1-ml fraction was collected by gel filtration chromatography.

### Mono Q ion exchange chromatography

Ion exchange chromatography was performed with a salt gradient using buffer A (20 mM Tris/HCl buffer, pH 7.4) and buffer B (1 M NaCl in buffer A, pH 7.4) at a flow rate 0.3 ml/min. Before loading the α1-PI containing fraction from the gel filtration chromatography, a MonoQ 5/50 GL column (GE Healthcare Life Science) was equilibrated with equilibration buffer (98.8% of buffer A/1.2% of buffer B). After injection of the diluted α1-PI sample with buffer A at a ratio of 1:4 to the ÄKTA 10s system, the column was washed using the equilibration buffer with five column volumes. The column-captured α1-PI was eluted and collected in 0.5-ml fractions by a stepwise gradient using 96.3% of buffer A and 3.7% of buffer B with 15 column volumes, followed by a linear gradient from 3.7 to 50% of buffer B with 14 column volumes.

### Desalting chromatography and concentration

The fractions containing α1-PI were desalted using three connected HiTrap Desalting 5 ml columns (GE Healthcare Life Science) with 100 mM sodium phosphate buffer at pH 7.2, including 150 mM NaCl at a flow rate 5 ml/min. According to the manufacturer’s instructions, the protein fraction was concentrated to 500 µl from 4 ml in a sample using Macrosep 30K red (Pall Life Sciences, Tokyo, Japan).

### Amino acid composition analyses of purified α1-PI

The α1-PI solution isolated by chromatography was hydrolyzed with 6 M HCl at 110°C for 20 h in evacuated sealed tubes. Amino acid analyses of protein hydrolysate were performed using the L-8900 amino acid analyzer. The amino acid composition of purified marmoset α1-PI was compared with the theoretical composition resulting from an amino acid composition search of the Swiss-Prot database.

### LC-MS/MS analyses for protein identification

An aliquot of the α1-PI fraction after desalting and concentrating was reduced by dithiothreitol, alkylated with iodoacetamide, and digested with trypsin for 3 h at 37°C in 50 mM ammonium bicarbonate. The resulting peptides were applied to Advance Nano LC coupled to LTQ equipped with a NANO-HPLC capillary column C18 (0.075 mm ID × 150 mm length, 3 µm particle size; Nikkyo Technos, Tokyo, Japan) using a linear gradient (48 min, 5–35% acetonitrile/0.1% formic acid) at a flow rate of 300 nl/min. The MS/MS data were searched against a database of *C. jacchus* (53016 sequences, significance threshold *P*<0.01) obtained from UniProtKB using MASCOT software (Matrix Science, U.K.).

### Production and purification of antibodies

Polyclonal antibodies against marmoset α1-PI were raised in five BALB/c mice and two Japanese White rabbits by repeated inoculation with 50 and 250 μg, respectively, of pure marmoset α1-PI emulsified in complete and incomplete Freund’s adjuvant. The raised antibodies were purified using a commercial kit (Proteus Protein G Mini Purification Starter Kit; Bio-Rad Laboratories, Hercules, CA) according to the manufacturer’s instructions. Purified antibodies from rabbits were used as capture antibodies, and purified antibodies from the mice were used as reporter antibodies.

### Collection of fecal and serum samples

Blood was collected from the femoral vein from eight healthy marmosets and seven WMS-affected individuals. The serum was separated and stored at −80°C until use. Fecal samples were collected from the same eight healthy animals and seven WMS-affected individuals. All of the naturally passed feces from 11:00 to 17:00 h were collected from each marmoset and placed in pre-weighed plastic tubes (Falcon 50 ml Polypropylene Conical Tube; Corning Life Sciences, Tewksbury, MA). All of the fecal samples were stored at −80°C until use. Collection of fecal and serum samples from WMS-affected marmosets was performed every 2 weeks, from pre-treatment to post-treatment. All WMS-affected marmosets were treated with tranexamic acid, as reported previously [14]. The serum concentration of Alb was measured using the Drychem 4000 system (FUJIFILM Co., Ltd., Tokyo, Japan). WMS-affected marmosets were defined as previously reported [14]: body weight (BW) < 325 g, hematocrit (Hct) < 32%, serum Alb < 4.4 g/dl, and serum matrix metalloproteinase 9 (MMP-9) > 27.9 ng/ml. On the other hand, healthy marmosets were defined as adult animals presenting with the following: BW > 350 g [6], Hct > 32% [25], serum Alb > 4.4 g/dl [25], and serum MMP-9 < 27.9 ng/ml [26]. In addition, levels of fecal calprotectin, a biomarker of marmoset chronic colitis [27] were <100 μg/g [28]. Animals that met the abovementioned criteria were used as healthy individuals (Table 1).

**Table 1:** Information of healthy marmosets used in the present study

	Sex	Age (years)	BW (g)	Hct (%)	Alb (g/dl)	MMP-9 (ng/ml)	Calprotectin (μg/g)
A	Male	5	488.9	46.5	5.5	17.3	41.19
B	Female	4	485.2	52.2	6.0	13.1	80.04
C	Female	6	425.9	48.2	4.9	16.4	11.11
D	Male	3	377.9	57.1	>6.0	24.3	34.20
E	Male	8	450.9	44.4	>6.0	10.3	21.51
F	Male	2	446.9	46.1	>6.0	3.1	0.00
G	Female	5	382.6	53.1	>6.0	8.0	0.00
H	Female	5	362.1	36.4	>6.0	16.9	0.00

### Extraction of fecal α1-PI

The following extraction protocol was used for the developed enzyme-linked immunosorbent assay (ELISA), and was modified from a procedure originally described by Melgarejo et al. [20]. Each frozen fecal sample was thawed and homogenized with a glass rod. Next, 100 mg of each wet sample was extracted with 10 ml phosphate-buffered saline (PBS) solution (0.05 M phosphate buffer, 0.1 M NaCl, pH 7.4, containing 5% newborn calf serum, and 0.01% thimerosal). After homogenization by vigorous shaking for 15 min at room temperature, the suspensions were centrifuged for 15 min at 1500×***g*** at 4°C. The supernatants were further centrifuged for 30 min at 12000×***g*** at 4°C. The final supernatants were transferred into other tubes and stored at −20°C until analyses. Extraction of fecal α1-PI for human α1-Antitrypsin ELISA kit (Immundiagnostik AG; Bensheim, Germany) was conducted following the manufacturer’s instructions. Each wet sample (15 mg) was extracted with 1.5 ml of the extraction buffer provided in the kit and stored at −20°C until subsequent analyses.

### Preparation of standards

A 250 mg/l solution of purified marmoset α1-PI was diluted to concentrations of 625, 208.3, 69.4, 23.1, 7.7, 2.6, and 0.0 μg/l, using PBS containing 0.1% polyoxyethylene sorbitan monooleate (Tween 80, Sigma–Aldrich Co. LLC, St. Louis, MO).

### ELISA

Each well of the microplate (NUNC-Immuno Plate; Thermo Fisher Scientific) was coated with 500 µg purified capture antibodies diluted in 100 µl of 50 mM sodium carbonate buffer (pH 9.6). The plate was incubated overnight at 4°C and then washed twice with PBS containing 0.05% Tween 20 (Sigma–Aldrich). Next, 100 µl PBS containing 0.2% bovine serum albumin was added to each well to block nonspecific antibody-binding sites. After incubation for 30 min at 37°C, the plate was washed three times with PBS/Tween 20. A volume of 100 μl of each of the seven standard solutions and 100 µl of diluted fecal extracts or diluted serum sample were added to the wells and incubated for 90 min at 37°C. The blank control consisted of 100 µl PBS/Tween 80. This blank control was used to confirm that the antibodies against α1-PI did not react with any other molecules including bovine serum albumin. After three washes with PBS/Tween 20, 400 ng purified reporter antibodies diluted in 100 µl PBS/Tween 80 were added to each well and incubated for 1 h at 37°C. The plate was again washed four times with PBS/Tween 20 and 1.6 ng peroxidase–conjugated goat anti-mouse IgG antibodies (Jackson Immuno Research Laboratories, Inc. West Grove, PA) in 100 µl PBS/Tween 80 were added to each well and incubated for 30 min at 37°C. After four washes with PBS/Tween 20, 100 µl of *o*-phenylenediamine dihydrochloride solution was added to each well. After 10 min of incubation, the reaction was stopped by adding 100 µl/well of 2 M sulfuric acid solution. The microplate was read at 490 nm using an automated microplate reader, and concentrations of serum and fecal α1-PI were calculated using the manufacturer’s software (Microplate Manager v6.2; Bio-Rad Laboratories, Hercules, CA, U.S.A.). The standard curve was based on a four parameter logistic fit using the formula y = (a − d)/(1 + (x/c)^b^) + d. The fecal α1-PI detection kit for humans (α1-Antitrypsin ELISA Kit; Immundiagnostik AG, Bensheim, Germany) was also evaluated for the detection of marmoset fecal α1-PI according to the manufacturer’s instructions, as follows. The standards, controls, and prediluted samples were added to the wells of a microplate coated with a high-affinity antibody against human α1-PI. After incubation and washing, a peroxidase–conjugated antibody against human α1-PI was added to each well. Tetramethylbenzidine was used as a peroxidase substrate and an acidic stop solution was added to terminate the reaction.

### Assay validation

The assay was validated by determining the lowest detectable concentration, dilutional parallelism (linearity), spiking recovery of added α1-PI (accuracy), and intra- and inter-assay coefficients of variation (reproducibility and precision, respectively). The validation procedures were performed separately for fecal extracts and serum samples. The lower detection limit was determined by setting up ten duplicates of samples with zero α1-PI (PBS/Tween 80), and calculating the concentration of α1-PI with an absorbance change equal to these ten duplicates. The linearity of the assay was determined by dilutional parallelism. Three serum samples from different marmosets were analyzed at dilutions of 1:5000, 1:15000, 1:45000, 1:135000, and 1:405000. The fecal samples from different marmosets were measured at dilutions of 1:300, 1:1000, 1:3000, and 1:9000. The percentage of bias after each dilution step was calculated from the observed/expected (O/E) ratios. For this, the observed α1-PI concentration (the value measured using the assay) was divided by the expected α1-PI concentration (the value calculated according to the dilution factor) and multiplied by 100 (O/E × 100). The accuracy of the assay was tested by spiking three serum samples and three fecal extracts each with known concentrations of purified marmoset α1-PI (for serum: 34.6, 22.6, 13.6, 7.6, 3.8, and 0.8 μg/l, and for fecal samples: 34.6, 22.6, 13.6, 7.6, 3.8, and 0.8 μg/g). Standard recovery after each spiking step was calculated from the obtained α1-PI concentration (the value measured using the assay), divided by the expected α1-PI concentration (the value calculated based on the added concentration of α1-PI) and multiplied by 100 (O/E × 100). The precision of the assay was determined by the evaluation of three serum samples and three fecal extracts ten times within the same assay on the same day (intra-assay variability). Reproducibility was determined by analyzing the same three serum samples and fecal extracts in ten and eight consecutive assay runs, respectively (inter-assay variability). Reproducibility and precision were calculated as coefficient of variation (CV% = Standard deviation (SD)/mean × 100%). The reference intervals for α1-PI in serum and fecal samples were calculated by mean ± 2SD.

### Statistical analyses

Statistical analyses were performed using commercially available software (GraphPad Prism 7; GraphPad Software Inc., San Diego, CA). Data from the values obtained from the developed ELISA and commercial kit using fecal samples were analyzed by the Mann–Whitney test. Data from the values for the α1-PI concentrations in serum and fecal samples of healthy and WMS-affected marmosets were also analyzed by the Mann–Whitney test. Data from the values for day-to-day variation along with the treatments were analyzed using repeated ANOVA. Values of *P*<0.05 were considered statistically significant.

## Results

### Purification of α1-PI from marmoset plasma

**Figure 1:**
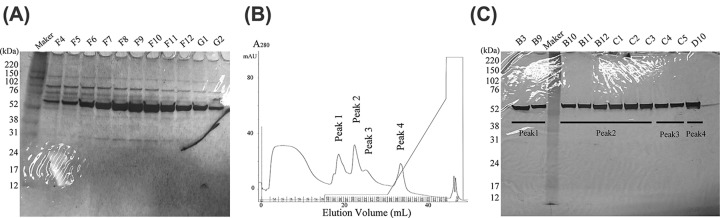
Visualization of purified α1-PI protein derived from marmoset plasma (**A**) Silver-stained SDS/PAGE for crude protein fractions obtained from the gel filtration chromatography. Each fraction consisted of multiple impurity proteins, including α1-PI. (**B**) Ion exchange chromatography using a MonoQ 5/50 GL column. The single peak fraction by the gel filtration containing α1-PI was separated into four peaks (peak 1–peak 4). (**C**) Silver-stained SDS/PAGE for α1-PI protein candidate fractions obtained from ion exchange chromatography. Lanes B3 and B9: peak 1, Lanes B10-C3: peak 2, Lanes C4-C5: peak 3, Lane D10: peak 4.

**Figure 2:**
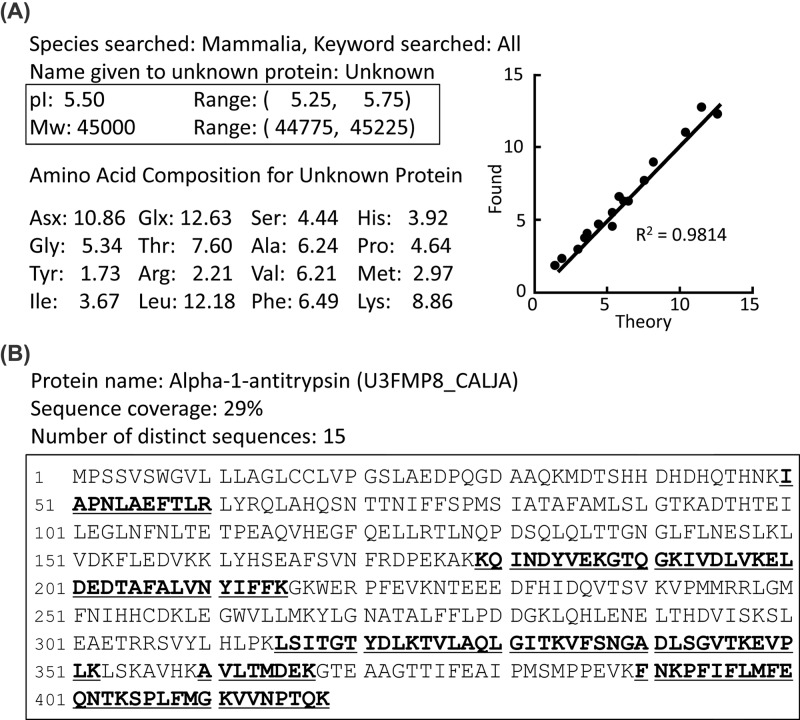
Identification of purified α1-PI protein in the database (**A**) Amino acid composition search using the Swiss-Prot Database. Comparison of amino acid composition ratio between theoretical and purified α1-PI. Data entry for the search is as follows: species, isoelectric point (pI) range, molecular weight range, and 18 amino acid molar percentage values. (**B**) Direct protein identification by LC-MS/MS analysis. Peptides identified as part of α1-PI protein by the Mascot database search are underlined.

The purity of marmoset α1-PI derived from plasma was increased by three steps: affinity chromatography using α1-antitrypsin select resin, gel filtration, and ion exchange chromatography. Although a single peak containing α1-PI on the chromatogram was observed at the gel filtration step, multiple bands were visualized on samples collected from the same peak area by sodium dodecyl sulfate/polyacrylamide gel electrophoresis (SDS/PAGE) with silver staining (Figure 1A). In total, four peaks (peak 1–peak 4) were observed from the collected samples on the chromatogram (Figure 1B) at the ion exchange chromatography step. At this step, a single band was detected in the purified samples of peak 1–peak 4 on SDS/PAGE with silver staining (Figure 1C). Among these, analyses of the amino acid composition showed that the sample peak 2 corresponded to the theoretical amino acid composition of marmoset α1-PI (Pearson correlation coefficient, r^2^ = 0.98) (Figure 2A). By contrast, the composition ratio of the samples peak 1, peak 3, and peak 4 differed more from that of the theoretical α1-PI. To clarify whether the purified sample included the candidate protein α1-PI, the trypsinized sample peak 2 was subjected to LC-MS/MS for protein identification. Only marmoset α1-PI (U3FMP8_CALIA) was identified, with 29% sequence coverage and 15 unique peptides (Figure 2B). Taken together, these results indicate that the sample peak 2 was the most purified marmoset α1-PI. Therefore, we decided to use this protein as the marmoset α1-PI reference sample for the following experiments.

### Standard curves and lower detection limit

Standard curves for the marmoset α1-PI ELISA were reproducible (see Supplementary Figure S1). The blank control exhibited similar or lower values compared with the 0.0 μg/l well, indicating that the antibodies against α1-PI did not react with any other molecules. The lower detection limit of the assay was 0.00000063 mg/ml in serum and 0.53 μg/g in the fecal samples.

### Linearity, accuracy, precision, and reproducibility of ELISA

The linearity of the developed ELISA was confirmed by dilutional parallelism. The O/E ratios for five serial dilutions of three serum samples ranged from 92.67 to 124.05% (mean ± SD: 110.41 ± 9.36%; Table 2). The O/E ratios for four serial dilutions of fecal extracts ranged from 79.96 to 124.24% (mean ± SD: 93.89 ± 12.27%; Table 3).

**Table 2:** Results for dilutional parallelism of serum samples for the marmoset α1-PI ELISA

	Dilution	Observed conc. (μg/l)	Expected conc. (μg/l)	O/E ratio (%)
Serum I	5000	408.43		
	15000	144.70	136.14	106.29
	45000	47.97	45.38	105.71
	135000	17.46	14.13	115.45
	405000	5.974	5.04	118.49
Serum II	5000	299.58		
	15000	92.54	99.86	92.67
	45000	40.25	33.29	120.91
	135000	12.95	11.10	116.75
	405000	4.17	3.70	112.69
Serum III	5000	285.25		
	15000	102.18	95.08	107.46
	45000	39.32	31.69	124.05
	135000	11.63	10.56	110.06
	405000	3.32	3.52	94.41

**Table 3:** Results for dilutional parallelism of fecal samples for the marmoset α1-PI ELISA

	Dilution	Observed conc. (μg/g)	Expected conc. (μg/g)	O/E ratio (%)
Feces I	300	0.59		
	1000	0.22	0.18	124.24
	3000	0.06	0.06	98.62
	9000	0.02	0.02	85.61
Feces II	300	0.76		
	1000	0.21	0.23	93.42
	3000	0.06	0.08	83.18
	9000	0.02	0.03	79.96
Feces III	300	1.30		
	1000	0.37	0.39	95.40
	3000	0.12	0.13	88.73
	9000	0.04	0.04	95.84

The accuracy of the developed ELISA was confirmed by spiking recovery. The O/E ratios for serum and fecal samples spiked with different concentrations of purified α1-PI ranged from 85.27 to 113.32% (mean ± SD: 95.44 ± 6.12%) for the serum samples (Table 4), and from 80.29 to 105.19% (mean ± SD: 90.46 ± 7.12%) for the fecal samples (Table 5).

**Table 4:** Results for spiking recovery of serum samples for the marmoset α1-PI ELISA

	Concentration added in μg/l	Observed conc. (μg/l)	Expected conc. (μg/l)	O/E ratio (%)
Serum I	0.00	3.58		
	0.80	4.97	4.38	113.32
	3.80	7.39	7.39	99.95
	7.60	9.96	11.18	89.03
	13.60	15.49	17.18	90.15
	22.60	24.33	26.18	92.93
	34.60	37.62	38.18	98.53
Serum II	0.00	4.97		
	0.80	5.56	5.77	96.46
	3.80	8.57	8.78	97.60
	7.60	11.46	12.57	91.16
	13.60	15.83	18.57	85.27
	22.60	25.54	27.57	92.65
	34.60	39.05	39.57	98.69
Serum III	0.00	5.77		
	0.80	6.40	6.57	97.39
	3.80	9.06	9.58	94.60
	7.60	12.47	13.37	93.30
	13.60	17.44	19.37	90.03
	22.60	26.54	28.37	93.57
	34.60	41.69	40.37	103.27

**Table 5:** Results for spiking recovery of fecal samples for the marmoset α1-PI ELISA

	Concentration added in μg/g	Observed conc. (μg/g)	Expected conc. (μg/g)	O/E ratio (%)
Feces I	0.00	0.03		
	0.80	0.66	0.83	80.29
	3.80	3.12	3.84	81.42
	7.60	6.37	7.63	83.56
	13.60	12.37	13.63	90.74
	22.60	21.19	22.63	93.64
	34.60	35.14	34.63	101.48
Feces II	0.00	0.01		
	0.80	0.69	0.81	85.71
	3.80	3.37	3.82	88.23
	7.60	6.35	7.61	83.53
	13.60	11.48	13.61	84.40
	22.60	20.99	22.61	92.83
	34.60	32.43	34.61	93.70
Feces III	0.00	0.32		
	0.80	0.96	1.12	85.73
	3.80	3.84	4.13	93.04
	7.60	7.10	7.92	89.58
	13.60	12.77	13.92	91.73
	22.60	23.74	22.92	103.57
	34.60	36.73	34.92	105.19

The precision of the developed ELISA was confirmed by intra-assay variability. The CVs for intra-assay variability were 12.11, 12.64, and 11.64% for the serum samples (Table 6), and 9.84, 7.88, and 4.99% for the fecal samples (Table 7).

**Table 6:** Precision and reproducibility of serum samples for the marmoset α1-PI

	Number of repeats	Mean (μg/l)	SD (μg/l)	CV (%)
Intra-assay variability				
Serum I	10	1205009.00	145898.44	12.11
Serum II	10	1246078.00	157520.87	12.64
Serum III	10	1043326.00	121493.88	11.64
Inter-assay variability				
Serum I	10	1412564.00	250417.74	17.73
Serum II	10	1440217.40	236474.78	16.42
Serum III	10	1180728.40	142272.85	12.05

**Table 7:** Precision and reproducibility of fecal extracts for the marmoset α1-PI

	Number of repeats	Mean (μg/g)	SD (μg/g)	CV (%)
Intra-assay variability				
Feces I	10	115.44	11.36	9.84
Feces II	10	198.47	15.64	7.88
Feces III	10	349.39	17.45	4.99
Inter-assay variability				
Feces I	8	135.54	22.05	16.27
Feces II	8	244.17	38.85	15.91
Feces III	8	414.81	56.34	13.58

The reproducibility of the developed ELISA was confirmed by inter-assay variability. The CVs for inter-assay variability were 17.73, 16.42, and 12.05% for the serum samples (Table 6), and 16.27, 15.91, and 13.58% for the fecal samples (Table 7).

### Reference intervals

The reference interval for serum α1-PI was 0.87–1.85 mg/ml, with a median of 1.27 mg/ml. The reference interval for fecal α1-PI was 0.53–395.58 μg/g, with a median of 108.76 μg/g.

### Developed ELISA versus the kit

**Figure 3:**
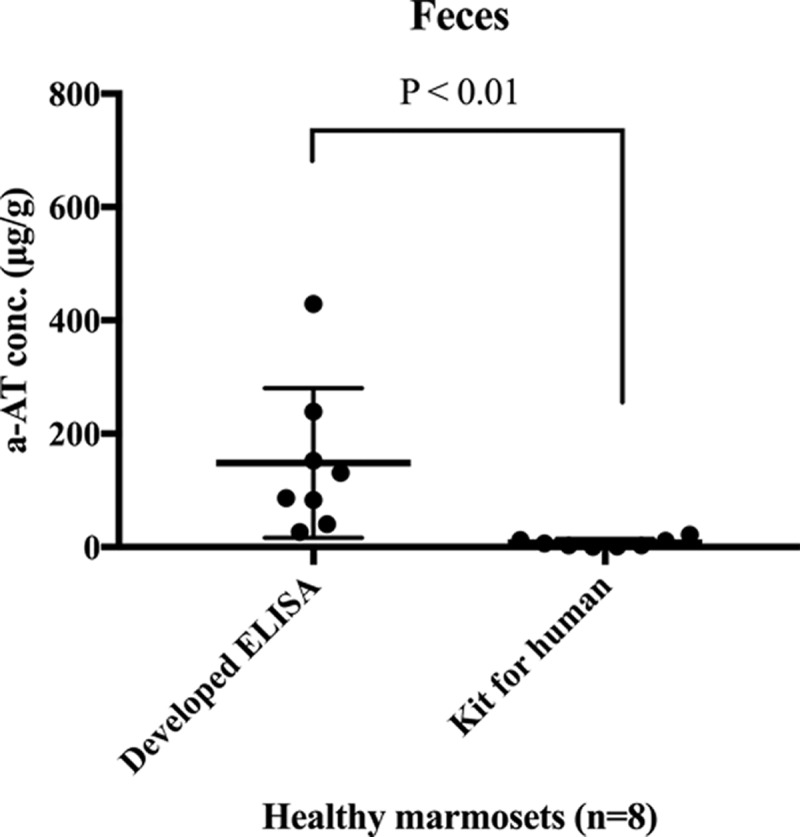
Comparison of the detection sensitivities for marmoset fecal α1-PI between our developed ELISA and a commercial kit for humans The values obtained from the developed ELISA were significantly higher than those from the commercial kit for humans (*P*<0.01).

The detection sensitivities of fecal α1-PI were compared between the developed ELISA and the commercial kit for humans. The α1-PI concentrations of fecal extracts from healthy marmosets were 148.41 ± 123.58 μg/g (mean ± SD) in the developed ELISA, whereas the concentrations of the same samples were 7.4 ± 6.7 μg/g in the kit. The values obtained from the developed ELISA were significantly higher than those from the commercial kit for humans (*P*=0.0002; Figure 3).

### Concentrations of α1-PI in serum and fecal samples from WMS-affected marmosets

**Figure 4:**
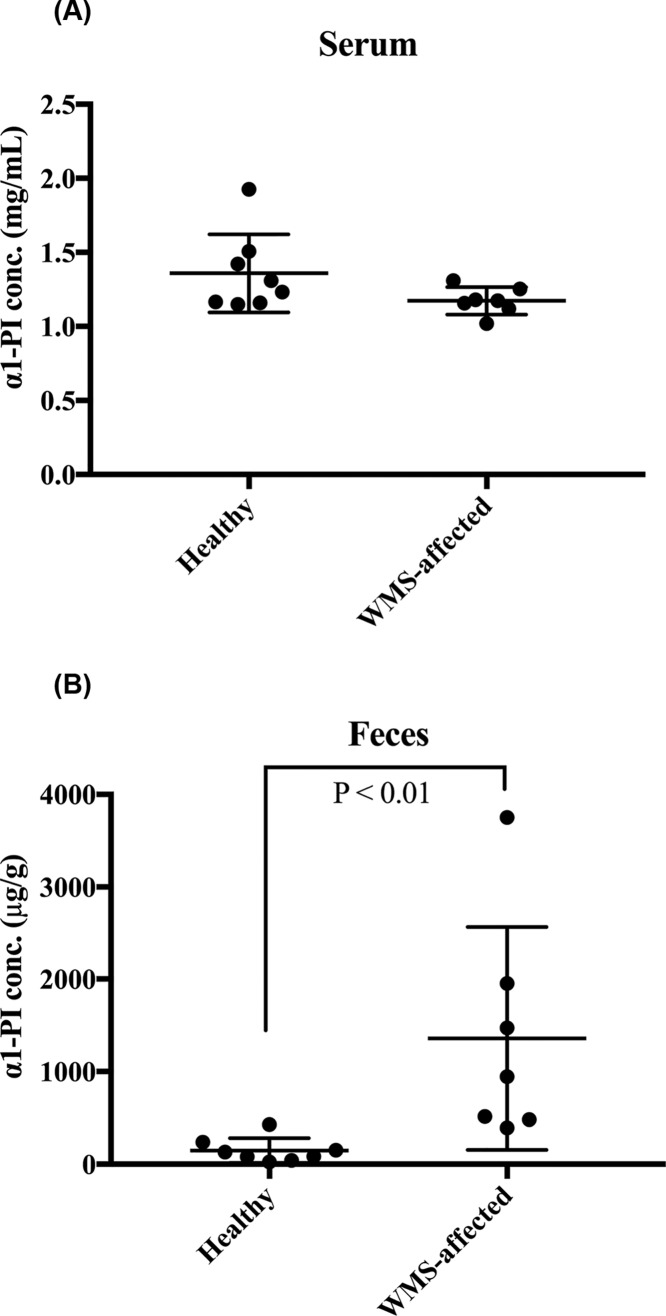
Comparison of concentrations of α1-PI in serum and fecal samples between healthy and WMS-affected marmosets (**A**) Serum concentrations of α1-PI. There was no significant difference in the α1-PI concentrations between healthy and WMS-affected marmosets. (**B**) Fecal concentrations of α1-PI. There was a significant difference in the α1-PI concentrations in the fecal samples between healthy and WMS-affected marmosets (*P*<0.01).

The α1-PI concentrations in serum and fecal samples from WMS-affected marmosets were 1.17 ± 0.86 mg/ml (mean ± SD) and 1357.58 ± 1113.32 μg/g before treatment. There was a significant difference in the α1-PI concentrations in the fecal samples between healthy and WMS-affected marmosets (*P*=0.0006; Figure 4A), whereas there were no significant differences in α1-PI concentrations in the serum between healthy and WMS-affected marmosets (*P*=0.1520; Figure 4B).

### Treatment effects on the fecal concentration of α1-PI and serum Alb concentration

**Figure 5:**
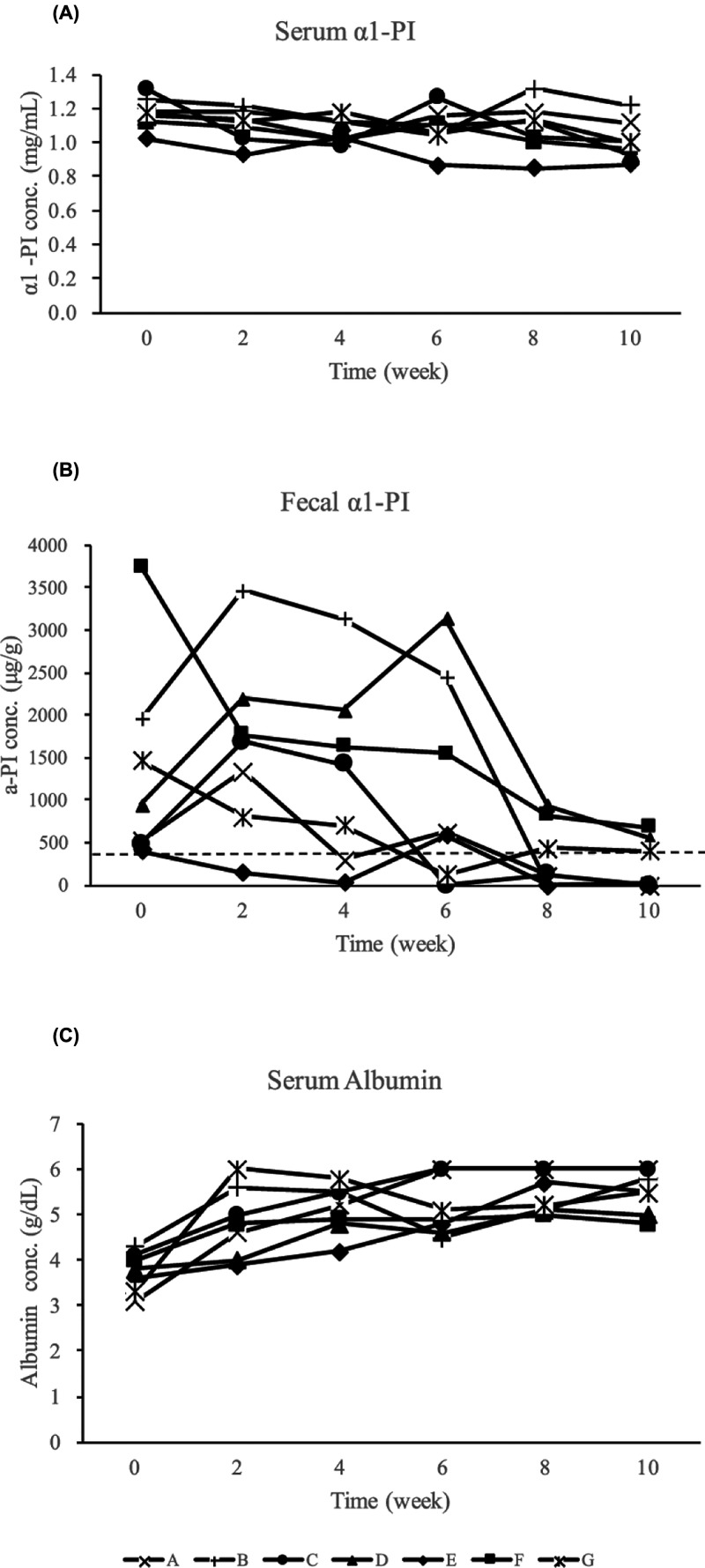
Changes in α1-PI concentrations in serum and fecal samples and of serum Alb concentration in WMS-affected marmosets and their treatment (**A**) Change in serum α1-PI concentration. The serum concentrations of α1-PI remained unaltered throughout the treatment. (**B**) The change in fecal α1-PI concentration. There was a significant treatment effect on the fecal concentration of α1-PI (*P*<0.05). The dashed line indicates the upper range of healthy marmosets. (**C**) The change in serum Alb concentration. A significant treatment effect was observed (*P*<0.01).

Changes in the α1-PI concentration in the serum and fecal samples obtained from WMS-affected marmosets, along with the treatments administered are presented in Figure 5. Although the concentrations of α1-PI in the serum remained unaltered throughout the treatment (F_5, 30_ = 2.766, *P*=0.0823; Figure 5A), there was a significant treatment effect on the concentration of α1-PI in feces (F_5, 30_ = 4.447, *P*=0.0227; Figure 5B). A significant effect of treatment on the serum levels of Alb was also observed (F_5, 30_ = 11.920, *P*= 0.0008; Figure 5C).

## Discussion

In the present study, purification of α1-PI from marmoset plasma was conducted differently than the method reported by Parambeth et al. [24]. The purity of marmoset α1-PI derived from plasma was increased using three different steps: affinity chromatography using α1- antitrypsin select resin, gel filtration, and ion exchange chromatography. The ion exchange chromatography step is unique and represents an important new methodology.

As described by Parambeth et al. [24], cross-immunoreactivity for α1-PI among species is limited; therefore, the development of specific antibodies for marmoset α1-PI was required. Using the purified α1-PI protein, two types of polyclonal antibodies specific for marmoset α1-PI were obtained. Using these two antibodies, an ELISA was developed to measure marmoset α1-PI in serum and feces. The lower detection limits of this assay were determined to be 0.00000063 mg/ml in serum and 0.53 μg/g in fecal samples. These values were acceptable because the reference intervals for serum and fecal samples from healthy marmosets were 0.84–1.85 mg/ml and 0.53–395.58 μg/g, respectively. The linearity of this assay was confirmed by dilutional parallelism studies. The O/E ratios for dilutional parallelism for serum and fecal samples had a mean ± SD of 110.41 ± 9.36 and 93.89 ± 12.27%, respectively, which was in accordance with data by Burke et al. [21]. The accuracy of this assay was confirmed by spiking recovery studies. The O/E ratios for spiking recovery for serum and fecal samples had a mean ± SD of 95.44 ± 6.12 and 90.46 ± 7.12%, respectively, which was in accordance with previous data [20,21]. The precision and reproducibility of this assay were confirmed by intra- and inter-assay variability tests, respectively. Intra- and inter-assay CV for serum α1-PI ranged from 11.64 to 12.64% and 12.05 to 17.73%, respectively. Intra- and inter-assay CV for fecal α1-PI ranged from 4.99 to 9.84% and 13.58 to 16.27%, respectively. These values were acceptable by reference to others [21,29] because a CV < 20% was described as adequate by Jacobson [30].

In the present study, the reference intervals of serum and fecal samples from healthy marmosets were calculated to be 0.87–1.85 mg/ml and 0.53–395.58 μg/g, respectively. The reference interval for serum α1-PI was comparable with the reference intervals of other species, for example, humans (1.10–2.10 mg/ml) [31], dogs (0.90–2.11 mg/ml) [20], and cats (0.25–0.60 mg/ml) [32], and (0.64–1.40 mg/ml) [21]. On the other hand, the reference interval for the fecal concentration of α1-PI was higher than that of other species, for example dogs (0.023–5.67 μg/g) [20], and (2.2–16.8 μg/g) [19] and cats (0.04–1.9 μg/g) [21]. Marmoset might be an animal with moderate intestinal protein loss even when healthy. Captive marmosets require high amounts of dietary protein [33], possibly because intravital protein has to be compensated. As reference intervals should be established from 40 to 120 samples from qualified individuals [34,35], the establishment of new reference intervals based on serum and fecal samples from a greater number of healthy marmosets are needed in the future. Under our review process, Parambeth et al*.* [36] reported the development of a sandwich ELISA for measuring marmoset α1-PI. They reported reference intervals of α1-PI in serum and fecal samples from healthy marmosets of 1.254–1.813 mg/ml and 11.5–42.2 μg/g, respectively. The reference interval for the serum concentration was similar to, but that for fecal concentration different from, that in our study; however, they also stated that the fecal concentration in marmoset α1-PI was much higher than those of other animals. These similarities support the validity of the data from both studies*.*

Because there is limited knowledge about the cross-immunoreactivity for α1-PI among species [37], we compared the values of the same fecal samples between our developed ELISA and a commercial kit for humans to ascertain the suitability of the kit for detecting marmoset α1-PI. There was a significant difference in the detection sensitivity of marmoset fecal α1-PI. The commercial kit had a 20-fold lower detection sensitivity compared with that of our developed ELISA. This comparison suggested little α1-PI cross-immunoreactivity between humans and marmosets. The concentrations of α1-PI in serum and fecal samples from seven WMS-affected marmosets were measured, and the concentration averages of the protein were 1.17 mg/ml and 1357.58 μg/g, respectively. Although there were no significant differences in the serum α1-PI concentration between healthy and WMS-affected marmosets, the α1-PI concentration was significantly higher in the fecal samples from WMS-affected marmosets than in samples from healthy individuals, suggesting that intestinal protein loss occurs in WMS. Before treatment, all fecal α1-PI concentrations in WMS-affected marmosets exceeded 395.58 μg/g, the upper range in healthy controls. The severity of the protein loss presumably depends on the extent of intestinal damage that has been sustained. Furthermore, similar widely ranging protein loss values have been reported among patients with Crohn’s disease [18]. The intestinal protein loss in WMS-affected marmosets was significantly reduced by treatment with tranexamic acid, which suppresses certain inflammatory cytokines by inactivating plasmin, a key enzyme in the fibrinolytic cascade [14]. This treatment might help in recovery of the damaged intestinal epithelia and decrease the amount of protein loss, resulting in a significant increase in the concentration of serum Alb. The intestinal protein loss appeared to be one of the mechanisms involved in hypoalbuminemia observed in WMS.

## Supporting information

**Supplementary Figure S1:** Representative standard curve for marmoset α1-PI ELISA.

## Abbreviations

Alb: albumin
BW: body weight
CV: coefficient of variation
ELISA: enzyme-linked immunosorbent assay
Hct: hematocrit
MMP-9: matrix metalloproteinase 9
O/E: observed/expected
PBS: phosphate-buffered saline
SD: standard deviation
SDS/PAGE: sodium dodecyl sulfate/polyacrylamide gel electrophoresis
WMS: wasting marmoset syndrome
α1-PI: α1-proteinase inhibitor
