# Supplementary material for: Measurement of the α1-proteinase inhibitor (α1-antitrypsin) of common marmoset and intestinal protein loss in wasting syndrome
Source: Biosci Rep. 2019 Jul 8;39(7):BSR20190562. doi: 10.1042/BSR20190562 (PMC6614574; doi:10.1042/BSR20190562)
Supplement: Supplementary file 1 [file bsr20190562_Supp1.pdf]

Supplementary material

Supplementary Figure

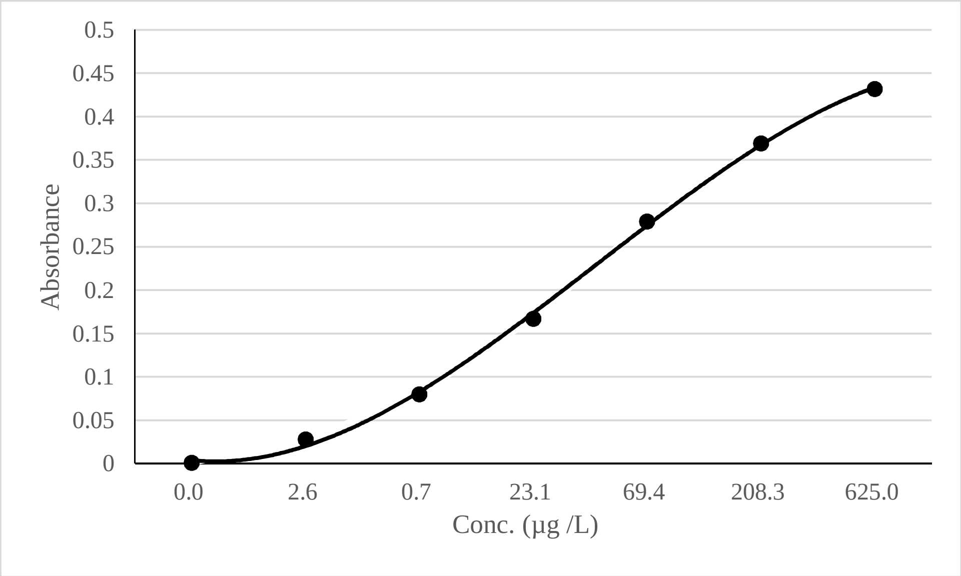

Fig. S1 Representative standard curve for marmoset α1-PI ELISA.

A typical standard curve for the marmoset α1-PI ELISA is shown.
